# Nutritional Management for Chronic Liver Disease—Current Trends and Future Prospects

**DOI:** 10.3390/nu17030579

**Published:** 2025-02-05

**Authors:** Takashi Himoto

**Affiliations:** Department of Medical Technology, Kagawa Prefectural University of Health Sciences, 281-1, Hara, Mure-cho, Takamatsu 761-0123, Kagawa, Japan; himoto@kagawa-puhs.ac.jp; Tel.: +81-87-870-1240

It has been well established that numerous nutritional factors, including macronutrients and micronutrients, are involved in the pathophysiology of chronic liver diseases [1,2,3]. Hepatitis C virus (HCV)-related chronic liver disease and nonalcoholic fatty liver disease (the term has since shifted to metabolic dysfunction-associated steatotic liver disease: MASLD [4]) are frequently associated with impaired glucose tolerance and dyslipidemia [5]. Cirrhotic patients often have sarcopenia [6]. Therefore, nutritional management is essential for the prevention or inhibition of progression to these liver diseases. Nutritional assessments are also required for nutritional intervention. Based on these backgrounds, this Special Issue, “Nutritional Management for Chronic Liver Disease” has been organized. This Special Issue aims to focus on current trends in nutritional management and novel hallmarks for nutritional assessment in chronic liver diseases. Five interesting articles which meet the focus, including three review articles and two original articles, have been published in this Special Issue.

One review article written by Komatsu et al. highlighted the clinical relevance of nutritional intervention in patients with adult-onset type II citrullinemia (CTLN2) [7]. The authors showed that patients with CTLN2 tend to lack sufficient energy intake and to have food preferences. CTLN2 patients preferred to have lower-carbohydrate, higher-fat, and higher-protein foods. An appropriate protein–fat–carbohydrate ratio has recently been proposed as a means of nutritional intervention to maintain the nutritional balance in such patients. However, CTLN2 patients have a clinical feature of lean MASLD. Therefore, nutritional intervention for MASLD may exacerbate the disease state in CTLN2 patients. On the other hand, the effect of medium-chain triglyceride (MCT) supplementation with minimal carbohydrate on hepatic encephalopathy has been exhibited in patients with CTLN2. The optimal dose of MCT should be determined for an improvement in hepatic encephalopathy.

Another review article submitted by Kosmalski et al. verified food-derived substances as therapeutic agents for MASLD using biochemical, nutritional, and histological parameters [8]. The gold standard treatment for MASLD has not yet been established thus far. Therefore, many kinds of foods, food components, and compounds have been administrated in MASLD patients as complementary treatments. This review article noted the efficacy of synbiotics, particularly Protexin, which reversed hepatic fibrosis in lean MASLD patients. The detailed mechanism by which Protexin ameliorates hepatic fibrosis should therefore be elucidated.

As a third review article, my colleagues and I provided an overview of the current trends in nutritional management for alcoholic liver diseases [9]. In our review article, the recommended levels of total energy and oral intake of macronutrients were provided for each category of alcoholic liver disease, including alcoholic hepatitis, alcoholic steatosis, and alcoholic liver cirrhosis. In addition, the involvement of micronutrients, including vitamins and trace elements, in the pathogenesis of alcoholic liver diseases was discussed. We also mentioned the clinical significance of treatment with vitamin D in patients with decompensated liver cirrhosis. The administration of vitamin D reversed the loss of skeletal muscle mass and muscle strength in such patients. The treatment appears to be promising and should be considered as an optional treatment in alcoholic liver cirrhosis associated with sarcopenia.

In the first original article published in this Special Issue, Kamada et al. investigated the correlations between the oral intake of nutrients and the severity of hepatic fibrosis in MASLD patients [10]. It was of interest that the dietary intake of mono-unsaturated fatty acid (mUSFA) was significantly lower in the group of advanced hepatic fibrosis than that of early hepatic fibrosis, whereas there was no significant difference in the intake of mUSFA between the MASLD and control groups. To the contrary, no significant difference in the oral intake of zinc was found between the groups of early and advanced hepatic fibrosis, although the intake was significantly lower in the group of MASLD patients than in the control group. Soluble dietary fiber was significantly higher in the group of MASLD than in the control group and lower in the group of advanced fibrosis than in the group of early fibrosis. The recommended dietary dose of soluble dietary fiber should be determined to prevent or inhibit the development of hepatic fibrosis in MASLD patients.

Another original article written by Tsukagoshi et al. highlighted the efficacy of geriatric nutritional risk index (GNRI) and prognostic nutritional index (PNI) in patients with hepatocellular carcinoma (HCC) who underwent hepatic resection [11]. GNRI is regulated by serum albumin level and actual/ideal body weight ratio. PNI is calculated using the serum albumin level and lymphocyte count in peripheral blood. The authors revealed that both GNRI and PNI were significantly associated with skeletal muscle index in their patients. It was notable that HCC patients with early recurrence had significantly lower GNRI and PNI than those without early recurrence. Moreover, these indices were significantly lower in HCC patients with extrahepatic recurrence than in those without extrahepatic recurrence. It is well recognized that microvascular invasion is closely associated with the recurrence of HCC. A histological examination of surgical specimens is required for the confirmation of microvascular invasion after hepatic resection. However, an assessment of GNRI and PNI enabled the authors to predict the recurrence of HCC under the preoperative condition without any histological findings. Nevertheless, the reason why these indices was closely correlated with the recurrence rate in HCC patients remains uncertain.

This Special Issue provides numerous novel insights into nutritional interventions for chronic liver diseases, including MASLD, alcoholic liver disease, CTLN2, and HCC. However, many issues of nutritional management remain unresolved in such patients. Further accumulation of evidence will be highly desirable. I sincerely appreciate the authors who submitted their attractive review articles and original articles to this Special Issue and the reviewers who gave valuable feedback to the authors.
